# Influence of genetic background on the occurrence of chromosomal rearrangements in *Saccharomyces cerevisiae*

**DOI:** 10.1186/1471-2164-10-99

**Published:** 2009-03-06

**Authors:** Emilie S Fritsch, Joseph Schacherer, Claudine Bleykasten-Grosshans, Jean-Luc Souciet, Serge Potier, Jacky de Montigny

**Affiliations:** 1 Laboratory of Molecular Genetics, Genomics and Microbiology, UMR7156, University of Strasbourg and CNRS, Strasbourg, France; 2 European Molecular Biology Laboratory, Heidelberg, Germany

## Abstract

**Background:**

Chromosomal rearrangements such as duplications and deletions are key factors in evolutionary processes because they promote genomic plasticity. Although the genetic variations in the *Saccharomyces cerevisiae *species have been well documented, there is little known to date about the impact of the genetic background on the appearance of rearrangements.

**Results:**

Using the same genetic screening, the type of rearrangements and the mutation rates observed in the S288c *S. cerevisiae *strain were compared to previous findings obtained in the FL100 background. Transposon-associated rearrangements, a major chromosomal rearrangement event selected in FL100, were not detected in S288c. The mechanisms involved in the occurrence of deletions and duplications in the S288c strain were also tackled, using strains deleted for genes implicated in homologous recombination (HR) or non-homologous end joining (NHEJ). Our results indicate that an Yku80p-independent NHEJ pathway is involved in the occurrence of these rearrangements in the S288c background.

**Conclusion:**

The comparison of two different *S*. *cerevisiae *strains, FL100 and S288c, allowed us to conclude that intra-species genomic variations have an important impact on the occurrence of chromosomal rearrangement and that this variability can partly be explained by differences in Ty1 retrotransposon activity.

## Background

DNA double strand breaks occur spontaneously or as a result of DNA damaging agents such as ionizing radiations or chemical reagents. If this damage is not properly repaired, it can lead to the occurrence of chromosomal rearrangements such as duplications, deletions and translocations, which can affect cell growth and survival. These rearrangements are key events in genome reshaping and evolution processes and many of the genomes sequenced to date show traces of these rearrangements [1,2]. In multicellular organisms, however, chromosomal rearrangements are often responsible for oncogenesis and for many human genetic diseases [3-5].

DNA double-strand break (DSB) repair mechanisms are therefore essential to each organism, since they preserve the integrity of the genome and prevent the deleterious effects of chromosomal rearrangements. These mechanisms can be classified in two distinct pathways: the homologous recombination (HR) pathway and the non-homologous end-joining (NHEJ) pathway. HR requires long homologous sequences for DSB repair whereas little or no homology is necessary for the NHEJ pathway.

**Figure 1:**
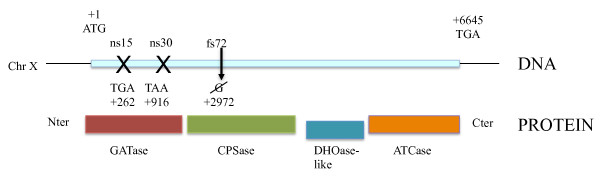
**The *ura2*_15,30,72 _allele and its corresponding multifunctional protein Ura2p**. ns15 and ns30 correspond to the positions of the non-sense mutations and fs72 to the position of the frameshift mutation. GATase stands for Glutamine AmidoTransferase, CPSase for carbamylphosphate synthetase, DHOase-like for dihydroorotase-like and ATCase for aspartyltranscarbamylase.

In order to select spontaneous chromosomal rearrangements, a genetic screening method based on a particular allele of the *URA2 *gene was developed (Figure 1) [6]. The *URA2 *gene is located on chromosome X and encodes a multifunctional protein, catalyzing the two first steps of the pyrimidine biosynthesis pathway and composed of glutamine amidotransferase (GATase), carbamoylphosphate synthetase (CPSase) and aspartyltranscarbamylase (ATCase) domains. The *ura2*_15,30,72 _allele has three point mutations located in its proximal region, which result in the loss of all the activities encoded by the *URA2 *gene. Both CPSase and GATase activities are compensated by two isoenzymes of the arginine biosynthesis pathway whereas the ATCase activity isn't. Thus the *ura2*_15,30,72 _strain is auxotrophic for uracil. However the ATCase activity can be reactivated by complex chromosomal rearrangements. This powerful screening tool can be used to perform *in vivo *experiments without the side-effects observed when using mutagenic agents or plasmid-encoded reporter genes.

In previous studies, rearrangements of several kinds were observed using this *URA2*-based screening method [6,7]. In the haploid FL100 context, three types of rearrangements leading to ATCase reactivation were detected: Ty1 insertions downstream of the last point mutation in the *ura2*_15,30,72 _allele, deletions of the region containing the three mutations, and duplications of the region encoding the ATCase followed by fusion with a new promoter sequence. Roelants *et al. *studied the ATCase reactivation resulting from Ty1 insertions and established that the transcription process is initiated in the LTR (Long Terminal Repeat) region of the Ty1 retrotransposon [8]. Deletions of the mutated region in the *ura2*_15,30,72 _allele were described by Welcker *et al. *and duplications of the ATCase region by Schacherer *et al. *[7,9].

An analysis of variations at the nucleotide level in some commonly used *Saccharomyces cerevisiae *strains was carried out by Schacherer *et al.*, who detected SNPs (Single Nucleotide Polymorphism) and deletions in various strains [10]. These sequence differences may have important effects on several biological pathways and phenotypes. A total number of 22,446 SNPs and 53 deletions were identified when the FL100 strain was compared to the S288c strain and the divergence observed between the two strains amounted to 0.21% [10]. To assess the effects of the genetic background on mutation rates and the type of chromosomal rearrangements, the *ura2*_15,30,72 _genetic screening was used to select spontaneous rearrangements in the S288c context. The results were compared with those previously obtained in the FL100 background [6,7].

Interestingly, in the S288c background, while duplications and deletions events were found to be responsible for the ATCase reactivation, no Ty1 insertions were observed. It was therefore concluded that the occurrence of chromosomal rearrangements is background-dependent. In addition, the occurrence of chromosomal deletions and duplications due to various recombination mechanisms was studied in haploid contexts. The impact of homologous recombination was tested by selecting revertants in a Δ*rad52 *strain. Since Rad59p plays an important role in single-strand annealing (SSA) processes between direct repeats, a Rad59p deficient mutant, Δ*rad59*, was constructed and selections of chromosomal rearrangements were performed in this background. The mutation rates and types of rearrangements observed in these two contexts were compared with a reference *ura2*_15,30,72 _strain. Inhibiting *RAD52*-dependent homologous recombination increased the deletion rate, whereas inactivation of the SSA pathway increased the duplication rate. Secondly, the effects of non-homologous end joining (NHEJ) were tackled by mutating *LIG4 *and *YKU80 *in the *ura2*_15,30,72 _background. The *LIG4 *mutation affected the mutation rates for both deletions and duplications, however there was no effect observed with *YKU80 *deletion. These results lead us to conclude that a Yku80p-independent NHEJ mechanism is responsible for the occurrence of chromosomal deletions and duplications.

## Results

### The genetic background affects the occurrence of chromosomal rearrangements

**Figure 2:**
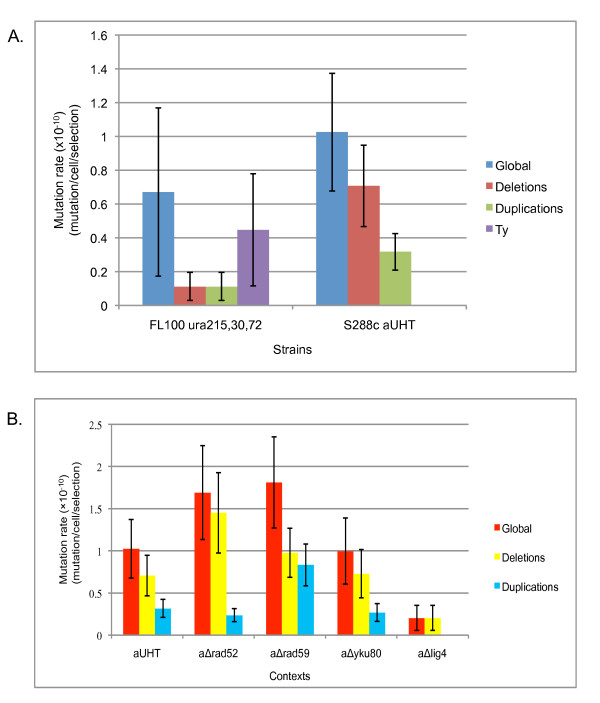
**Mutation rates in the different genetic contexts**. A. Global mutation rate and rates specific for every type of rearrangements in FL100 and S288c backgrounds. B. Mutation rates for the different recombination mutants in the S288c background. Error bars give the standard deviations.

In previous studies, selections were carried out in a FL100 *ura2*_15,30,70 _context. Among the chromosomal rearrangements leading to ATCase reactivation in FL100, Ty1 insertions usually account for 66%, deletions for 17% and duplications for 17% (Figure 2A) [6,7].

In this study, a S288c background (aUHT strain) was used to select chromosomal rearrangements and a comparison of these results with those previously seen in FL100 allowed us to establish whether the genetic background affects the mutation rate and the type of rearrangements. Using the *ura2*_15,30,72 _genetic screening method, 155 selections, amounting to 3.78 × 10^11 ^cells were performed and 29 independent revertants were obtained. The mutation rate determined using a maximum-likelihood method was 1.025 × 10^-10 ^mutations/cell/selection (confidence interval CI: 0.676 × 10^-10^; 1.373 × 10^-10^) (Figure 2A and Table 1).

**Table 1:** Number of revertants obtained in the studied contexts and the corresponding mutation rates.

Strains	No. of revertants or ATCase reactivation events (mutation rate × 10^-10^) (95% confidence interval)		
	
	Global	Deletion	Duplication
aUHT	29 1.025 (0.676; 1.373)	20 (69%) 0.707 (0.466; 0.947)	9 (31%)0.317 (0.210; 0.426)

aΔ*rad52*	21 1.69 (1.133; 2.247)	18 (86%) 1.45 (0.974; 1.932)	3 (14%) 0.237 (0.158; 0.314)

aΔ*rad59*	37 1.81 (1.27; 2.35)	20 (54%) 0.977 (0.685; 1.269)	17 (46%) 0.832 (0.584; 1.081)

aΔ*yku80*	22 0.997 (0.604; 1.39)	16 (73%) 0.728 (0.441; 1.015)	6 (27%) 0.269 (0.163; 0.375)

aΔ*lig4*	9 0.204 (0.055; 0.353)	9 (100%) 0.204 (0.055; 0.353)	0

The mutation rates (mutations/cell/selection) were determined using the maximum-likelihood method described by Lea and Coulson (1949). The 95% confidence limits were calculated using Student's *t*-test.

Among the 29 revertants leading to an ATCase reactivation, 20 (69%) carried deletions and 9 (31%) duplications. The boundaries of deletions were determined and microhomologies ranging from 1 to 11 bp in size were observed (see Additional file 1).

Contrary to what was observed in FL100 by Roelants *et al.*, no reactivation by Ty1 insertions was found to occur in the S288c background although a greater number of deletions and duplications were observed in that background [6]. These findings suggest that either there may be fewer Ty1 retrotransposons in S288c than in FL100 or that Ty1 retrotransposons may be less active in S288c than in FL100, and may thus be less likely to insert upstream from the ATCase region leading to a reactivation of the activity. Schacherer *et al. *showed that the proportion of Ty1 elements seems to be similar in these two backgrounds, hence the difference of Ty1 insertions may be explained by a variation of retrotransposons activity between FL100 and S288c [10].

### The Ty1 activity is lower in the S288c than the FL100 background

Paquin and Williamson established that the temperature affects the rate of Ty transposition [11]. They showed that the rate of transposition increases at temperatures below 30°C, which is the optimal growth temperature for *Saccharomyces cerevisiae*. In order to determine if the absence of Ty1 insertions in S288c resulted from a decrease in Ty1 transposition activity, selections were performed at 25°C. Twenty-four selections amounting to 1.04 × 10^11 ^cells were performed at 25°C and we obtained 16 independent revertants. The mutation rate was determined to be: 3.70 × 10^-10 ^mutations/cell/selection (CI: 2.12 × 10^-10^; 5.27 × 10^-10^). This mutation rate was 3.6 times higher than at 30°C. In addition, at 25°C, four Ty1 insertions (25%) were detected, whereas none were observed at the optimal growth temperature. These results led to the conclusion that the Ty1 retrotransposons are active in the S288c background but that they show lower rates of activity than those observed in the FL100 background.

The mechanisms leading to the occurrence of chromosomal deletions and duplications were studied using S288c at 30°C, in order to neglect Ty1 insertions and focus on the deletions and duplications events. Daley and Wilson showed that deletions can occur due to HR, SSA and NHEJ mechanisms, according to the DNA overhang length [12]. Schacherer *et al. *proposed that the mechanism involved in the occurrence of genic duplications is a HR-dependent retroposition mechanism [9]. Lastly Koszul *et al. *suggested that break induced replication (BIR) might lead to the appearance of segmental duplications [13]. In this study we decided to focus on the impact of homologous recombination and non-homologous end joining processes in the occurrence of deletions and duplications.

### Involvement of a homologous recombination mechanism in the occurrence of chromosomal deletions and duplications

#### The deletion rate increases in the Δ rad52 context

The first target used to investigate the impact of homologous recombination on the selection of chromosomal rearrangements was *RAD52*. Rad52p is a major component of HR: it binds to ssDNA as multimers and acts as a mediator allowing Rad51p to bind to ssDNA. It also plays a role in Rad51p-independent homologous recombination mechanisms. A deletion in *RAD52 *results in defective HR-associated double-strand break repair processes.

In this context, 83 selections corresponding to 2.4 × 10^11 ^cells were performed leading to the occurrence of 21 independent revertants. The mutation rate in the aΔ*rad52 *context was 1.69 × 10^-10 ^mutations/cell/selection (CI: 1.13 × 10^-10^; 2.25 × 10^-10^), as shown in Table 1.

Among the 21 characterized revertants, 3 (14%) were duplications and 18 (86%) deletions (Table 1 and Figure 2B) with microhomologies ranging from 1 to 11 bp (see Additional file 1). There were no significant differences between the duplication rates, but the deletion rate was found to be twice as high as that recorded in the reference strain. This indicates that defective homologous recombination leads to an increased number of deletions.

#### The occurrence of duplications is repressed in RAD59

Using the approach described above, we used a strain mutated for a gene involved in homologous recombination and notably in the SSA pathway [14]. Among the various candidates available, *RAD59 *was chosen. Rad59p is a protein which contributes to DNA DSB repair by annealing complementary single-strand DNA. In the aΔ*rad59 *strain, 130 selections corresponding to 2.88 × 10^11 ^cells were carried out and 37 revertants were isolated. The mutation rate in the aΔ*rad59 *context was found to be 1.81 × 10^-10 ^mutations/cell/selection (CI: 1.27 × 10^-10^; 2.35 × 10^-10^), and no statistical differences were observed in comparison with the reference strain.

The 37 revertants could be identified as 20 deletions (54%) and 17 duplications (46%). The microhomologies observed at the deletion boundaries ranged from 1 bp to 11 bp in size (see Additional file 1). The duplication rate was 2.5 times higher in this context than in the reference aUHT strain. This result is statistically significant whereas no significant difference was observed for the deletion rates. This allows us to conclude that Rad59p restrains the appearance of duplications.

### Involvement of NHEJ in the occurrence of deletions and duplications

In order to test the impact of NHEJ on the occurrence of deletions and duplications, two deletion mutants, aΔ*yku80 *and aΔ*lig4*, were constructed in the *ura2*_15,30,72 _background. Yku80p is a subunit of the Ku complex involved in telomere maintenance and in DNA binding at DSB sites, and Lig4p is the DNA ligase required for the NHEJ mechanism, along with the cofactors Lif1p and Nej1p.

#### The occurrence of deletions and duplications is Yku80p-independent

In the aΔ*yku80 *context, 135 selections corresponding to 2.7 × 10^11 ^cells were performed and 22 independent revertants were thus obtained. The mutation rate in the aΔ*yku80 *context was 0.997 × 10^-10 ^mutations/cell/selection (CI: 0.604 × 10^-10^; 1.39 × 10^-10^) (Figure 2B and Table 1). No significant differences were observed in this respect between aΔ*yku80 *and the reference strain.

Among the 22 characterized revertants, 6 carried duplications (27%) and 16 deletions (73%) with microhomologies ranging from 2 to 11 bp in size (see Additional file 1). These results are in agreement with the data obtained for the reference strain. It was therefore concluded that in our system, deletions and duplications can occur in a Yku80p-independent context.

#### A Δlig4 context leads to a decrease of DNA repair

In the aΔ*lig4 *background, 151 selections corresponding to 3.7 × 10^11 ^cells were performed and 9 independent revertants were isolated. The mutation rate was found to be 0.204 × 10^-10 ^mutations/cell/selection (CI: 0.055 × 10^-10^; 0.353 × 10^-10^), which corresponds to a statistically significant 5-fold decrease. Molecular characterization of the 9 revertants showed that all the reversion events resulted from deletions with microhomologies ranging from 1 to 11 bp in size (see Additional file 1). When *LIG4 *is inactivated, a decrease of efficient repair is observed. Moreover since the deletion rate was found to be 3.4 times lower in this context, it was concluded that Lig4p has an impact on the occurrence of deletions. Moreover, no duplications could be selected, which suggests that the Lig4p inactivation also inhibits the appearance of duplications in the *ura2*_15,30,72 _system.

## Discussion

It was recently established that DNA sequence variations frequently occur among *S*. *cerevisiae *strains [10,15]. These sequence differences may have important effects on the phenotypes [16-18]. However, little is known so far about the impact of genetic variations on the occurrence of chromosomal rearrangements.

Using the *URA2*-based genetic screening, we investigated the impact of the genetic background on the types of chromosomal rearrangements and the mutation rates.

### Different genetic backgrounds lead to a variation of the type of selected chromosomal rearrangements

To test the impact of the genetic background, two *S*. *cerevisiae *strains, S288c and FL100 were compared in terms of rate of occurrence for various rearrangements such as deletions, duplications and Ty1 retrotransposon insertions. Interestingly, in the S288c context, no Ty1 retrotransposition insertions were selected, whereas these rearrangements account for 2/3 of the selected events in FL100. These findings point to the conclusion that either there may be fewer Ty1 retrotransposons in S288c than in FL100 or that they show lower levels of Ty1 activity.

Intra-species variations of retrotransposons location and number have been well described [19,20]. Nevertheless, since the proportion of Ty1 elements seems to be similar in FL100 and S288c backgrounds, a difference of Ty1 activity can probably account for the differences between the Ty1 insertions [10].

Paquin and Williamson reported that Ty transposition is temperature-sensitive and that this process is enhanced at temperatures below the optimal growth temperature [11]. The activity of the retrotransposons in the S288c background was therefore tested by selecting revertants at 25°C. At that temperature, 4 Ty1 insertions leading to ATCase reactivation were selected. This finding shows that Ty1 retrotransposons are active in the S288c background but their activity is probably decreased in that background compared to FL100.

In this study, intra-species variations were found to occur as regards the occurrence of chromosomal rearrangements between the S288c and the FL100 strains, and this variability was attributed to differences in the activity of the Ty1 elements. In addition, the regulation of the Ty1 retrotransposition might differ between the two strains. For instance, it has been established that *S*. *cerevisiae *undergoes transcriptional and post-transcriptional controls which limit transposition in a copy-number dependent manner [21,22]. Differences in these control mechanisms might also explain the transposition variations observed between the two strains. Further studies on the FL100 strain are now required to determine how the activity of Ty1 retrotransposons is regulated and establish their exact locations.

### Non-Homologous End Joining contributes to the occurrence of deletions

Contrary to what occurs in higher eukaryotes, where non-homologous recombination processes predominate, the main DNA double-strand break repair pathway in yeast is the homologous recombination pathway [23]. It was therefore proposed to investigate the contribution of HR on the appearance of chromosomal deletions.

In a Δ*rad52 *context, the deletion rate was found to be twice as high as in the reference strain, which suggests that the occurrence of deletions is favoured when *RAD52*-dependent homologous recombination is inactivated. This finding is in agreement with previously observed results in the FL100 background [7].

To form deletions by homologous recombination, a homologous sequence at least 60 bp long is required [24]. However, since no sequence homologous to the ATCase-coding domain is present upstream from the *URA2 *gene, no ATCase reactivation is possible via the HR recombination pathway. In addition, since microhomologies (1 to 11 bp) were detected at the junctions of the deletions in every context, it was concluded that a mechanism of non-homologous recombination was probably responsible for the occurrence of the deletions.

When *LIG4*, the ligase required for NHEJ to occur, was mutated, we observed a decrease in deletion rate. However, when *YKU80*, which is also involved in NHEJ, was mutated, no differences were observed in comparison with the reference strain. Thus these results suggest that the repair mechanism involved in the occurrence of deletions is independent of Yku80p.

Boulton and Jackson established that two different NHEJ pathways can be distinguished in *S*. *cerevisiae *[25]. One of these pathways is accurate and Ku-dependent, whereas the other one is error-prone, Ku-independent and involves short homologies. In addition, it has been suggested that the two NHEJ pathways might lead to different chromosomal rearrangements in *S*. *cerevisiae *[26]. Given these observations, it seems likely that the occurrence of deletions in the *ura2*_15,30,72 _context may be attributable to a Ku-independent NHEJ mechanism. These deletions would result from a DSB followed by a NHEJ repair involving short sequence homologies. In addition, when HR is inactivated, the rates of HR and NHEJ seem to be unbalanced and repair by NHEJ is favoured, thus leading to a higher rate of deletions.

We should mention that a compensatory mutation would have the same impact, since it would mask the effect of a *YKU80 *deletion. Nevertheless, this hypothesis is very unlikely since the Y16546 strain, from which the aΔ*yku80 *background derives, was also used for functional analysis of a *YKU80 *deletion in other studies, showing one of the expected phenotype: the presence of short telomeres [15].

### The occurrence of duplications depends on NHEJ

Rad59p is involved in homologous recombination and notably in the SSA mechanism, which involves short homologous sequences [27]. In a Δ*rad59 *context, a 2.5-fold increase in the duplication rate was observed, which suggests that inactivation of the SSA pathway may favour the occurrence of duplications.

On the other hand, in a Δ*lig4 *context, the fact that no duplications were selected suggested that the duplication rate was greatly impaired. However, when *YKU80 *was mutated, no significant differences were observed in the duplication rates. It was therefore concluded that a Ku-independent NHEJ mechanism is involved in the occurrence of duplications in our system. Duplications may possibly result from two DSBs events followed by a NHEJ repair process occurring between two microhomologies at the DNA ends.

## Conclusion

In this study, we investigated the mechanisms involved in the occurrence of chromosomal rearrangements and we tested the influence of intraspecies variability.

The results indicate that a non-homologous end joining mechanism independent of the Ku complex may be responsible for the occurrence of deletions and duplications in S288c. This repair mechanism is highlighted in absence of HR and SSA, which suggests that in a wild-type strain, a balance between the various mechanisms exists to maintain the integrity of the genome. The occurrence of deletions in the S288c context was similar to those previously suggested in a FL100 background [7]. Although phenotypic differences and sequence variations can be observed from one strain to another, the use of major repair pathways such as HR and NHEJ remains unchanged.

By contrast, no Ty1 insertions were detected in S288c whereas these are major events contributing to ATCase reactivation in the FL100 background. This variation may be attributed to a difference in Ty1 activity rather than a difference between the mechanisms involved, since the repair mechanisms seems to be the same between FL100 and S288c when using the *ura2*_15,30,72 _system. Further analysis of the activity of retrotransposons and their regulation in the FL100 background and a comparison of the activity in S288c may lead to confirmation of this hypothesis.

## Methods

### Yeast strains and media

Yeast cells were grown at 30°C in liquid or solid (2% agar) yeast peptone dextrose (YPD) and supplemented yeast nitrogen base (YNB). All the strains used in this study are listed in Table 2. The BY4711 (*α trp1*Δ*63*) and BY4714 (*a his3*Δ*200*) strains were crossed and the diploid was sporulated. One of the spores with a *MATa URA2 his3*Δ*200 trp1*Δ*63 *genotype was transformed with the *ura2*_15,30,72 _allele to obtain the aUHT strain (*a ura2*_15,30,72 _*his3*Δ*200 trp1*Δ*63*). Single deletion mutants were constructed by crossing the reference strain *a ura2*_15,30,72_*his3*Δ*200 trp1*Δ*63 *(aUHT) with strains from the EUROSCARF deletion collection (Y16546 (Δ*yku80*), Y11781(Δ*lig4*), Y10540 (Δ*rad52*) and Y13756 (Δ*rad59*)). The diploids were then sporulated and one spore with the expected phenotype was selected (Table 2).

**Table 2:** Yeast strains used in this study.

**Strains**	**Genotype**	**Origin**
**BY4711**	*MATα trp3*Δ63	Brachmann *et al*., 1998 [31]

**BY4714**	*MATa his3*Δ200	Brachmann *et al*., 1998

**Y16546**	BY4742 *MATα his3*Δ*1 leu2*Δ*0 lys2*Δ*0 ura3*Δ*0 YMR106c::KANMX4*	EUROSCARF collection Brachmann *et al*., 1998

**Y11781**	BY4742 *MATα his3*Δ*1 leu2*Δ*0 lys2*Δ*0 ura3*Δ*0 YOR005c::KANMX4*	EUROSCARF collection Brachmann *et al*., 1998

**Y13756**	BY4742 *MATα his3*Δ*1 leu2*Δ*0 lys2*Δ*0 ura3*Δ*0 YDL059c::KANMX4*	EUROSCARF collection Brachmann *et al*., 1998

**Y10540**	BY4742 *MATα his3*Δ*1 leu2*Δ*0 lys2*Δ*0 ura3*Δ*0 YML032c::KANMX4*	EUROSCARF collection Brachmann *et al*., 1998

**aUHT**	*MATa ura2*_15,30,72 _*his3*Δ*200 trp1*Δ63	This study

**aΔ*yku***	*MATa ura2*_15,30,72 _*his3*Δ*200 trp1*Δ*63 YMR106c::KANMX4*	This study

**aΔ*lig4***	*MATa ura2*_15,30,72 _*his3*Δ*200 lys2*Δ*0 YOR005c::KANMX4*	This study

**aΔ*rad52***	*MATa ura2*_15,30,72 _*his3*Δ*200 lys2*Δ*0 YML032c::KANMX4*	This study

**aΔ*rad59***	*MATa ura2*_15,30,72 _*his3*Δ*200 trp1*Δ*63 YDL059c::KANMX4*	This study

The diploid obtained from a cross between BY4711 and BY4714 was sporulated and one spore was transformed with the *ura2*_15,30,72 _allele thus leading to the aUHT strain. All the BY strains are direct descendants of the FY2 strain which itself is directly descended from S288c [31].

Single deletion mutants were constructed by crossing aUHT with strains from the EUROSCARF deletion collection (Y16546 (Δ*yku80*), Y11781(Δ*lig4*), Y10540 (Δ*rad52*) and Y13756 (Δ*rad59*)). The diploids were then sporulated and one spore with the expected phenotype was selected.

### Selection of Ura+ mutants

One isolated colony was grown in 300 μl YPD medium overnight, plated on YPD medium and incubated at 30°C for 4–5 days. The cells were then harvested, resuspended in 1.5 ml water and spread on supplemented YNB without uracil. The cells were then incubated at 30°C thus allowing the growth of spontaneous Ura^+ ^revertants. In order to determine the total number of cells plated, a 100 μl aliquot of the culture was diluted 10^6 ^times, plated on YPD medium and incubated at 30°C. This procedure corresponds to one independent selection. The same procedure was used to perform selections at 25°C.

### Mutation rate determination

The mutation rates (mutations/cell/selection) were determined using the maximum-likelihood method described by Lea and Coulson (1949) [28]. The 95% confidence limits were calculated using Student's *t*-test.

### Southern blot analysis

Genomic DNA from *S. cerevisiae *was prepared as described by Hoffman and Winston [29]. DNA digestions were performed with the *Bam*HI restriction endonuclease (ROCHE) as described by the manufacturer. DNA digestions were migrated in a 1% agarose gel by electrophoresis and transferred onto a Hybond N^+ ^membrane. Digoxygenin-labeled DNA probes were prepared using a DNA labeling kit (ROCHE) and detection was then carried out using a NBT/BCIP colorimetric method (ROCHE).

The type of chromosomal rearrangements present in the various selected revertants was determined by performing a *Bam*HI restriction pattern analysis based on Southern blot hybridization. A DNA probe specific to the ATCase domain was used and a single 6.8 kb band corresponding to the size of this domain was observed in the *ura2*_15,30,72 _strain. A modified restriction profile was detected in all the selected revertants. In the case of a deletion or a Ty1 retrotransposon insertion, a single band differing in size from 6.8 kb was observed, whereas in the case of a duplication, two bands were detected: one corresponding to the *ura2*_15,30,72 _allele and one corresponding to the duplicated ATCase coding region. We then discriminated between deletions and Ty1 insertions events using a PCR approach.

### PCR amplification, DNA sequencing and sequence analysis

The primers used for PCR amplification and sequencing were chosen on the basis of the published genomic sequence of S288c. DNA fragments were obtained by performing PCR amplification using *Taq *DNA polymerase from MP Biomedicals, and the PCR conditions used were those described by the manufacturers. The Ty1 insertions in the *URA2 *coding sequence were characterized by performing PCR using two retrotransposon Ty1 LTR specific primers (sense and reverse) and a specific primer for the coding sequence of the ATCase. This allowed us to detect sense and reverse Ty1 insertions. The deletions were detected by performing primer-walking PCR to determine the presence or absence of a PCR product between close primer pairs. This made it possible to define the boundaries of the deletions.

The PCR products were purified using MicroSpin S400 (GE Healthcare). DNA sequencing was then performed on the purified fragments as described by Sanger *et al. *[30]. The sequencing was carried out using AmpliTaq FS DNA polymerase and BIGDYE TM terminators. Sequence reactions were analyzed with an Applied Biosystems 373XL sequencer.

## Authors' contributions

ESF did the main experiments and drafted the manuscript. CBG contributed to the construction of the S288c *ura2*_15,30,72 _strain and was involved in the manuscript revision. JS, JLS, SP, JdM were involved in manuscript editing and data analyses. All authors read and approved the manuscript.

## Supplementary Material

Additional file 1 **The different microhomologies observed at the boundaries of the deletions in this study.** The data represents the short repeated sequences observed for the deletions events. The coordinates of the boundaries are defined according to the +1 ATG of the *URA2 *gene. The putative ATG positions and proteins length were defined by *in silico *analysis. Click here for file
