# Supplementary material for: Influence of genetic background on the occurrence of chromosomal rearrangements in Saccharomyces cerevisiae
Source: BMC Genomics. 2009 Mar 6;10:99. doi: 10.1186/1471-2164-10-99 (PMC2674068; doi:10.1186/1471-2164-10-99)
Supplement: Additional file 1 — The different microhomologies observed at the boundaries of the deletions in this study. The data represents the short repeated sequences observed for the deletions events. The coordinates of the boundaries are defined according to the +1 ATG of the URA2 gene. The putative ATG positions and proteins length were defined by in silico analysis. [file 1471-2164-10-99-S1.doc]

| **Boundaries 5' *URA2* (included)** | **Boundaries 3' *URA2* (included)** | **Deletion Length (nt)** | **Microhomology at the junction** | **Microhomology length** | **Position of the putative ATG** | **Putative protein length (aa)** | **Context** |
| --- | --- | --- | --- | --- | --- | --- | --- |
| **-626** | +3346 | 3972 | G | 1 | +3352 | 1097 | aUHT |
| **+50** | +3999 | 3948 | TCTAC | 5 | +1 | 898 | aUHT |
| **+56** | +4005 | 3948 | GGTGA | 5 | +1 | 878 | aUHT |
| **+138** | +4006 | 3267 | CTGGTGAA | 8 | +1 | 925 | aUHT |
| **+196** | +3416 | 3219 | TCCATCTTATG | 11 | +1 | 1141 | aUHT |
| **-512** | 3347 | 3858 | GAAAGAATTGA | 11 | +3352 | 1097 | aUHT |
| **-717** | +3275 | 3992 | - | 0 | +3301 | 1114 | aΔ*rad52* |
| **-645** | +3231 | 3876 | A | 1 | +3262 | 1127 | aΔ*rad52* |
| **-710** | +3267 | 3977 | T | 1 | +3301 | 1114 | aΔ*rad52* |
| **-504** | +5394 | 5898 | ACC | 3 | +5407 | 412 | aΔ*rad52* |
| **+177** | +3370 | 3192 | TGAATCT | 7 | +1 | 1150 | aΔ*rad52* |
| **+196** | +3416 | 3219 | TCCATCTTATG | 11 | +1 | 1141 | aΔ*rad52* |
| **-696** | +4023 | 4719 | - | 0 | +4156 | 829 | aΔ*rad59* |
| **-573** | +3341 | 3914 | - | 0 | +3352 | 1097 | aΔ*rad59* |
| **115** | +3179 | 3063 | G | 1 | +1 | 1193 | aΔ*rad59* |
| **222** | +3595 | 3372 | C | 1 | +1 | 1090 | aΔ*rad59* |
| **-643** | +3100 | 3743 | T | 1 | +3184 | 1153 | aΔ*rad59* |
| **-66** | +4054 | 4120 | G | 1 | +4156 | 829 | aΔ*rad59* |
| **228** | +4096 | 3867 | ATTG | 4 | +1 | 925 | aΔ*rad59* |
| **+56** | +4005 | 3948 | GGTGA | 5 | +1 | 878 | aΔ*rad59* |
| **-352** | +3891 | 4243 | GCCTTA | 6 | +3988 | 885 | aΔ*rad59* |
| **+177** | +3370 | 3192 | CTGAATCT | 8 | +1 | 1150 | aΔ*rad59* |
| **-512** | 3347 | 3858 | GAAAGAATTGA | 11 | +3352 | 1097 | aΔ*rad59* |
| **-512** | 3347 | 3858 | GAAAGAATTGA | 11 | +3352 | 1097 | aΔ*rad59* |

| **Boundaries 5' *URA2* (included)** | **Boundaries 3' *URA2* (included)** | **Deletion Length (nt)** | **Microhomology at the junction** | **Microhomology length** | **Position of the putative ATG** | **Putative protein length (aa)** | **Context** |
| --- | --- | --- | --- | --- | --- | --- | --- |
| **-435** | +4090 | 4525 | TT | 2 | +4156 | 829 | aΔy*ku80* |
| **+43** | +3992 | 3948 | AATGG | 5 | +1 | 898 | aΔy*ku80* |
| **+56** | +4005 | 3948 | GGTGA | 5 | +1 | 878 | aΔy*ku80* |
| **+138** | +4006 | 3267 | CTGGTGAA | 8 | +1 | 925 | aΔy*ku80* |
| **+139** | +3579 | 3439 | TGGTGAATT | 9 | +3583 | 1020 | aΔy*ku80* |
| **-512** | 3347 | 3858 | GAAAGAATTGA | 11 | +3352 | 1097 | aΔy*ku80* |
| **+196** | +3416 | 3219 | TCCATCTTATG | 11 | +1 | 1141 | aΔy*ku80* |
| **159** | +3241 | 3081 | G | 1 | +1 | 1187 | aΔ*lig4* |
| **401** | +2853 | 2451 | TGA | 3 | +3052 | 1197 | aΔ*lig4* |
| **144** | +3583 | 3438 | GTT | 3 | +1 | 1068 | aΔ*lig4* |
| **-741** | +2925 | 3666 | CGATTT | 6 | +3052 | 1197 | aΔ*lig4* |
| **+177** | +3370 | 3192 | CTGAATCT | 8 | +1 | 1150 | aΔ*lig4* |
| **+139** | +3579 | 3439 | TGGTGAATT | 9 | +3583 | 1020 | aΔ*lig4* |
| **-512** | 3347 | 3858 | GAAAGAATTGA | 11 | +3352 | 1097 | aΔ*lig4* |

**Additionnal file 1**: The different microhomologies observed at the boundaries of the deletions in this study.

The coordinates of the boundaries are defined according to the +1 ATG of the *URA2* gene. The putative ATG position and protein length were defined by *in silico* analysis.
